# Functional symmetry and reproducibility of the evolutionary process

**DOI:** 10.18699/vjgb-26-32

**Published:** 2026-04

**Authors:** S.I. Bartsev

**Affiliations:** Institute of Biophysics of the Siberian Branch of the Russian Academy of Sciences, Krasnoyarsk, Russia Siberian Federal University, Krasnoyarsk, Russia

**Keywords:** reproducibility of the evolutionary process, equifinality of evolutionary outcomes, functional symmetry, heuristic neural network model, functional complexity, воспроизводимость эволюционного процесса, эквифинальность эволюционных исходов, функциональная симметрия, эвристическая нейросетевая модель, функциональная сложность

## Abstract

The question on the reproducibility of evolutionary processes is primarily of fundamental importance; however, with the development of methods for modeling evolutionary processes on computer multilevel models, an answer to this question is necessary to clarify the status of the predictions obtained. Experimental obtaining of ensembles of evolutionary outcomes for subsequent statistical processing on real biological systems seems to be impracticable. At the same time, the results obtained on multilevel computer models are difficult to interpret due to their complexity and the dependence of modeling results on a variety of parameters. This work is aimed at identifying common properties of evolving systems using a simple heuristic model based on transparent general principles and ideas about the key properties of biological systems that are important for the evolutionary process. Agents undergoing evolutionary changes are recurrent neural networks with a well-defined structure, a given function, and a specific rule for modifying the structure in the direction of maximum fitness. A separate instance of a neural network formed during the evolutionary process is called neural network model object (NNMO). Computational experiments have been carried out to generate ensembles of NNMO structures performing a given function, and the patterns of NNMO distribution in the structural space have been analyzed. This analysis confirms the presence of functional symmetry in the structure of NNMOs performing the same function. An assessment of the stability and reproducibility of individual evolutionary trajectories has been carried out. It is shown that under certain constraints leading to a reduction of the complexity of the NNMO structure (analogous to a narrow environmental specialization), the final NNMO structures may be close, but not identical. This suggests an inaccurate reproduction of the evolution of the structure with functional equivalence. Nevertheless, it can be argued that in the general case, the very ability for evolutionary change is possible with the redundancy of the potential complexity of the structure over the functional complexity and automatically entails a multiplicity of evolutionary outcomes based on the fact that the same function can be implemented by different, but functionally invariant structures.

## Abstract (ru)

Вопрос о воспроизводимости эволюционных процессов имеет в первую очередь фундаментальное значение, однако с развитием методов моделирования эволюционных процессов на компьютерных многоуровневых моделях ответ на этот вопрос необходим для прояснения статуса получаемых прогнозов. Экспериментальное получение ансамблей эволюционных исходов для последующей статистической обработки на реальных биологических системах представляется неосуществимым. В то же время прогнозы, сгенерированные многоуровневыми компьютерными моделями, вследствие их сложности и зависимости результатов моделирования от множества параметров с трудом поддаются интерпретации. Данная работа направлена на выявление общих свойств эволюционирующих систем с помощью простой эвристической модели, построенной на прозрачных общих принципах и представлениях о ключевых свойствах биологических систем, значимых для эволюционного процесса Агенты, претерпевающие эволюционные изменения, являются рекуррентными нейронными сетями с четко определенной структурой, заданной функцией и определенным правилом модификации структуры в направлении максимальной приспособленности. Отдельный экземпляр нейронной сети, формируемой в ходе эволюционного процесса, назван нейросетевым модельным объектом (НМО). В работе проведены вычислительные эксперименты по генерации ансамблей структур НМО, выполняющих заданную функцию, и проанализированы закономерности распределения НМО в структурном пространстве. Этот анализ подтверждает наличие функциональной симметрии структуры НМО, выполняющих одну и ту же функцию. Оценены устойчивость и воспроизводимость индивидуальных эволюционных траекторий. Показано, что при определенных ограничениях, приводящих к редукции сложности структуры НМО (аналог – узкая экологическая специализация), финальные структуры НМО могут быть близки, но не идентичны. Это позволяет говорить о неточном воспроизведении эволюции структуры на фоне функциональной эквифинальности. Тем не менее можно утверждать, что в общем случае сама способность к эволюционным изменениям реализуется при избыточности потенциальной сложности структуры над функциональной сложностью и автоматически влечет за собой множественность эволюционных исходов, основанную на том, что одна и та же функция может реализовываться различными, но функционально инвариантными структурами.

## Introduction

The extent of our misunderstanding of life can be seen from the abundance of problems associated with it. One of the key scientific problems of biology is the problem of predictability and/or possible equifinality of biological evolution.

According to M. Eigen, “every single system that has emerged as a result of mutation and selection is unpredictable in terms of its structure; nevertheless, the inevitable result is always the process of evolution – this is the law. …The optimizing process of evolution is in principle inevitable, although the choice of a particular path is not deterministic” (Eigen, 1971).

At the same time, there is a certain parallelism in the evolution of various species and genera, which found its expression in the law of homological series in hereditary variability by N.I. Vavilov (Meyen et al., 1977). If there is some structural similarity in systems with the same function, but formed along different evolutionary trajectories, then we can talk about the equifinality (Meyen, 1974; Meyen et al., 1977) of evolution in a certain sense.

The question of whether evolution follows a deterministic path or can follow alternative trajectories attracts many researchers (Povolotskaya, Kondrashov, 2010; Lobkovsky et al., 2011; Orgogozo, 2015; Xue et al., 2017). Currently, there are contradictions between some theoretical concepts and experimental data on the reproducibility of evolutionary trajectories.

For example, the paper (Lobkovsky, Koonin, 2012) notes that with a high intensity of mutations, evolution can follow different trajectories on the fitness landscape, but, ultimately, they all converge at a single peak corresponding to the most adapted structure of the evolving biological system. And the paper (Orgogozo, 2015) considers the possibility of predicting genotype by phenotype, i. e. the author admits the existence of a one-to-one mapping of the genotype into the phenotype, which means the uniqueness of the evolutionary outcome, that is, the similarity of the genotype with the same phenotypeThere are experiments in favor of the uniqueness of evolutionary outcomes (Weinreigh et al., 2006; Dickins, Nekrutenko, 2009; Meyer et al., 2012), but there is evidence (Poelwijk et al., 2007; Dunham et al., 2009; Kvitek, Sherlock, 2011; Podgornaia, Laub, 2015; Starr et al., 2017) demonstrating the existence of several endpoints of evolution

The complexity of research in this area is due to the fact that direct research of the evolutionary processes of biological systems in nature encounters three main obstacles: (A) the uniqueness of evolutionary outcomes, which does not allow using comparative analysis of biological structures; (B) the characteristic time of evolutionary changes, which usually exceeds the life span of the researcher many times; (C) the extreme complexity of real biological objects.

A possible approach to overcoming the stated obstacles immediately suggests itself: this is mathematical modeling and its special kind – computer modeling

Computer modeling makes it possible to overcome the first and second of these obstacles: the researcher can obtain ensembles of model evolutionary trajectories to which statistical data processing can be applied to identify common patterns of evolutionary processes. But the status of the results of computer modeling depends on how well it is possible to cope with the complexity of the biological system and provide the adequacy of its description. There are two main obstacles.

First, when modeling real events, models are needed to be as close in complexity as possible to real systems, but here we face the curse of dimensionality – the number of fitting parameters in conditions of uncertainty about the exact type of functional dependencies makes it impossible to determine them due to a lack of evolutionary data.

Secondly, there are doubts (Beckage et al., 2011; Garte et al., 2025) about the possibility of constructing adequate mathematical models of biological systems in general and evolutionary models in particular. The authors (Garte et al., 2025) note that “biological concepts resist clear definitions amenable to mathematical treatment. These include being alive and being an individual, as well as agency, inheritance, intelligence, sentience, and cognition” and on this basis they argue that the next “paradigm shift will require not just new mathematical tools but a new scientific EPISTEMOLOGY”, that is, ways of representing knowledge. At the same time, in their opinion, “choosing which data to pay attention to is far more valuable than collecting large amounts of data”.

One of the ways to solve the equifinality problem is to carefully analyze the theoretical arguments and experimental data available in the press in favor of a particular position.

Another way is to get expected and unexpected answers based on the development of initial principles and general ideas about the evolution of life. The study presented here is based on previously obtained results (Bartsev, Bartseva, 2002, 2006, 2010; Bartsev, Baturina, 2019).

## Methods and materials

Since the approach to studying the properties of biological evolution used in this paper is quite unusual from the point of view of traditional molecular biology, it seems necessary to focus on the methodological foundations of the proposed approach.

Models and model objects are tools of scientific research, starting with Galileo, who, technically unable to directly study the free fall of bodies, derived formulas for the kinematics of equidistant motion using a physical model – balls rolling down an inclined chute. In the future, the following definition will be useful to us: “If similarity can be established between two objects in at least one specific sense, then between these objects there is original-model relationship” (Lerner, 1972).

The paper will use heuristic models (Von Neumann, 1966), which, unlike traditional models, are not aimed at modeling (describing) specific real systems, but are abstract models designed to identify convenient concepts, widely applicable principles, and build a general theory.

Von Neumann justified the usefulness of moving towards heuristic model objects as follows: “Appealing to the organic, living world does not help us greatly, because we do not understand enough about how natural organisms function. We will stick to automata which we know completely because we made them…” (Von Neumann, 1966).

Some ways of setting the problem with this approach can be distinguished from Von Neumann’s reasoning: “Automata theory seeks general principles of organization, structure, language, information, and control. Many of these principles are applicable to both natural and artificial systems, and so a comparative study of these two types of automata is a good starting point. Their similarities and differences should be described and explained. Mathematical principles applicable to both types of automata should be developed.

...The question that one can then hope to answer, or at least investigate, is: What principles are involved in organizing these elementary parts into functioning organisms, and what are the essential quantitative characteristics of such organisms?” (Von Neumann, 1966).

The requirements for a heuristic model of an evolving biological system have long been formulated by J. Bernal: “Biology differs methodologically from other natural sciences in that the focus is primarily on the functioning and evolution of systems. Structure is important here only in relation to function and origin...” (Bernal, 1968, p. 112).

Bernal’s identification of functioning as a special characteristic of living beings is consistent with the approach of N. Rashevsky and R. Rosen, who considered the organism as a “set or system of functional mappings” (Rosen, 1958; Rashevsky, 1968, p. 63).

Practically any study of living objects is first the identification of functional patterns such as “stimulus–response” or “impact–response”, and then the definition of “mechanisms” that provide the implementation of this function (catalysis, inheritance, locomotion, recognition, etc.). The mechanism of the system is considered disclosed if it is described in terms of interactions of the parts that make up this system.

Formally, the mechanism is revealed by decomposing the original function F(x) into simpler, basic functions {gi} implemented by the corresponding subsystems {Si} – the elements of the structure. Coupling coefficients {aij} describe the interaction (relationship) between the elements of the structure and actually define the structure of the system.

It is important to emphasize that the decomposition procedure corresponds to the network description, where {Si} are the nodes of the network, and {aij} are the coupling coefficients between the nodes. The network description has long been widely used in various fields of biology: from the description of the structure-property correlation of a chemical compound (Golovanov et al., 1998) to metabolic pathways, protein-protein interactions, genetic networks, the nervous system, food chains of ecosystems, etc. (Albert et al., 2000; Amaral et al., 2000; Edelman, Gally, 2001; Strogatz, 2001; Dunne et al., 2002; Sole, 2002; Stumpf et al., 2008; De Las Rivas, Fontanillo, 2010).

To describe a biological system, a network model must be able to evolve. An evolutionary process will be understood as a change in the structure of a biological system in accordance with some functional criterion of optimality (Eigen, 1971; Forst et al., 1995; Schuster, 1996).

An analysis of existing formal models shows that networks of formal neurons, the so-called neural networks (NNs), are the most adequate objects for the purposes of this study. The advantage of NNs as a heuristic model of evolution is that it is easy for them to determine the FUNCTION, highlight the STRUCTURE and start the learning process, the formal description of which does not differ in key features from the process of biological EVOLUTION

**Fig. 1.**
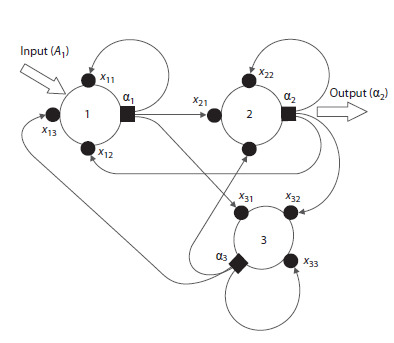
A fully connected neural network of three neurons: 1 – input neuron; 2 – output neuron; 3 – associative (hidden) neuron, which is not directly connected to the input and output

In this study, fully connected recurrent NNs were used, which can function in a stream of discrete events. Figure 1 illustrates a digraph of the simplest NN of 3 neurons, describing the ways in which signals are received and exchanged.

Formulas describing the functioning of the NN have the form:

**Formula. 1.**
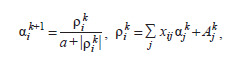
Formula. 1.

where α k i is the output signal of the i-th neuron at the k-th moment of time; ρ k i is the weighted sum of the output signals of the neurons received by the i-th neuron at the k-th moment of time, plus the external input signal Ak i , received by the i-th input neuron at the k-th moment of time; xij is the matrix of weighting coefficients (synapses); a is the coefficient specifying the steepness of the activation function.

From the formula, it can be seen that the output signal of each neuron (its level of excitation) at each time depends on the state of the NN (and the external signal for the input neurons) at the previous time

The modification of the NN structure was carried out by a random search algorithm, which was performed as follows: (1) a random increment was added to each synapse of the current NN, and two modified NNs were immediately created, differing in that the same modulo random increments were added to the corresponding synapses with different signs; (2) all three NNs – the initial one and its two modified versions – were started to function in a stream of events with a duration of 100 symbols, and for each NN, the total goal function (loss function) for this fragment was estimated – the sum of the squares of the differences between the outputs of the NN and the required response; (3) based on the results of the comparison, the NN with the minimum goal function was chosen, then it was compared with the required level of accuracy, and if it exceeded it, the algorithm was restarted from step 1. Formally, this procedure corresponds to random mutational substitutions

In order for the NN to act as a model of the evolutionary process, it is necessary to clearly state what the similarities are between it and a biological species in the process of evolution. The key components of the evolutionary process are heredity, variability, and natural selection, i. e. survival of the fittest. The measure of fitness for biological systems is difficult to deduce (Garte et al., 2025), but it is obvious that this measure should take into account the ability to reproduce and successful competition for resources.

In the course of natural selection, the fittest survive, therefore, to model natural selection, a measure of the fitness of one or another variant of the structure of NN is necessary. For neural networks, the goal function or loss function acts as a measure of fitness, which determines which variant of the NN structure will continue to evolve (learn). In other words, the goal function integrally includes an assessment of success in reproduction and competition for resources. In our case, the carrier of heredity is the structure of interneuronal connections, which is inherited by the next generation of the NN. The variability of the structure is provided by random increments of synapses, which leads to a variation in the quality of functioning of the neural network, i. e. a change in its adaptability to the environment in which it learns. We consider the evolutionary process in principle; the implementation features in the material are nuances.

**Fig. 2.**
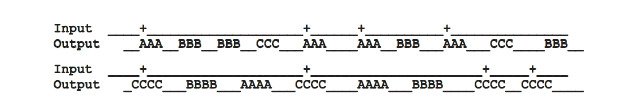
Fragments of the input and output sequences for functions A3 and C4. The “+” sign in the output sequence indicates the “correct” operation of the NN.

During learning (evolution) and during functioning, a continuous, quasi-random sequence consisting of signals was applied to the input of the NN: “pause” – (00), A – (10), B – (01) and C – (11), where the numbers in parentheses indicate the presence or absence of a signal at the corresponding input neurons. The random parameters of the sequence were: the type of signal that will be received at the input and the duration of the pause between the signals. According to the training pattern, the neural network should output (11) if the input receives the “correct” signal, and (00) otherwise. The sequences may differ in the number of time cycles provided to the neural network for processing the input signal (3 or 4), which is indicated in the function designation (Fig. 2).

Despite their simplicity, such sequences are suitable for simulating a wide class of processes in biological systems.

Any biological system exists in a continuous flow of time and discrete flow of events; therefore, discrete states in which the system remains during specific time interInput vals can be distinguished: the enzyme can be in a free or substrate-bound state; the glycolytic pathway can be in the mode of either glycolysis or gluconeogenesis, which, in particular, is determined by the state of the key enzyme phosphofructokinase/fructose-biphosphatase; the animal may be in a state of sleep, food intake, prey pursuit, etc. W. Ashby (1956) drew attention to the fact that a change in the types (patterns) of animal behavior can be described as a change in the states of a finite automation. In this case, the algebra of regular events (for example, Hopcroft et al., 2007, chapter 3) can formally describe the environment (and its complexity) in which the automation “lives” and the neural network “evolves”.

Since computational experiments were aimed at identifying common patterns of the evolutionary process, ensembles of neural networks performing the same function were created during the experiments. For brevity, a separate instance of a trained neural network will be called a neural network model object (NNMO)

The initial structure of the NNMO (a matrix of weighting coefficients) was set by a random number generator modulo close to 0. Training ended when a given level of the goal function was reached, corresponding to the complete absence of errors in recognizing stimuli in the input stream. That is, all the trained instances implemented the required function equally well. The generation and reduction of the complexity of NNMOs was carried out using the original Lazarus (Object Pascal) program.

For visualization and statistical analysis, the structure of the NNMO was represented as a point in the space of weighting coefficients; the Euclidean distance between points corresponding to different NNMOs can be taken as a measure of the proximity of their structures. In addition to the distribution of NNMO structures in the structural space, the paper evaluated the divergence of trajectories when starting from the same initial state. Visualization was performed using the original program in the Scilab 6.1.1 environment.

## Results

It should be immediately noted that the network description assumes the presence of addition and multiplication operations to describe the interaction of elements and their mutual influence on each other. But the specific values of a sum or product can be obtained in an infinite number of ways if we use non-integers. Hence, it’s possible to assume, or rather say with certainty, that in the general case, the maximum values of the fitness function can be achieved in different ways, i. e. by different functionally invariant structures. The results of computational experiments have confirmed this assumption.

Note that the minimum number of neurons in the NNMOs of the considered configuration, which provides the required quality of functioning, is 6, with two input neurons, two output neurons, and two associative or hidden neurons. Attempts to use NNs with fewer neurons made in this and earlier works (Bartsev, Bartseva, 2010) were unsuccessful. Thus, the minimum space of NNMO structures performing the specified functions is 36-dimensional. The localization of NNMO structures was visualized by projecting a 36-dimensional space into a 3-dimensional one, with various combinations of weight coefficient numbers being selected as the coordinates of the projection space.

**Fig. 3.**
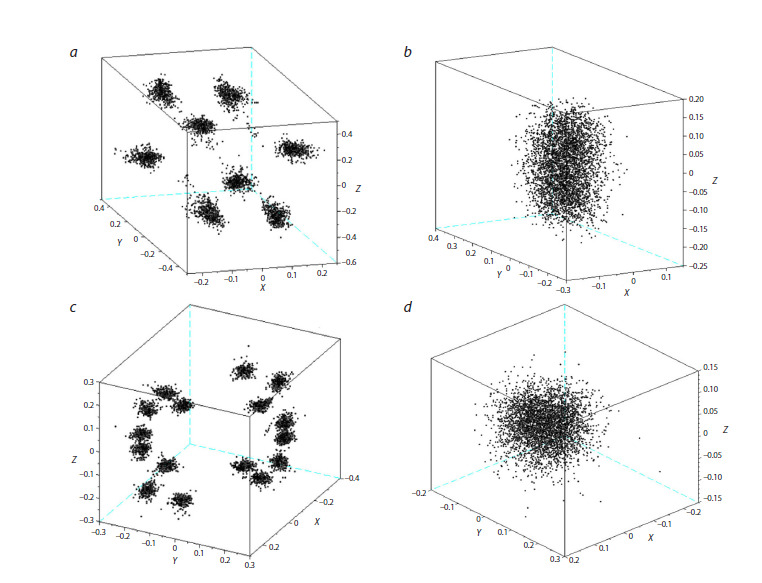
The location of NNMOs structures with the function A3 (a, b) and C4 (c, d). The XYZ coordinates for (a) and (c) correspond to the numbers of synapses 25/26/31. For (b) and (d) the coordinates are 9/12/22.

It was previously shown that the structures of NNMOs in space are not distributed randomly (diffusely), but form clusters (Fig. 3). Since the points correspond to NNMO structures that perform the same function, we can talk about the presence of functional symmetry, that is, the preservation of an invariant (function) under certain transformations (displacements in the space of NNMO structures).

Note that displacements within clusters correspond to local symmetry, and jumps from cluster to cluster correspond to discrete transformations of permutation and sign change (Bartsev, Bartseva, 2006, 2010). The number of clusters N for NNMOs with an odd activation function is determined by the formula: N = 2K K!, where K is the number of associative neurons. The power of 2 describes the number of sign-changing combinations, and the factorial describes the number of permutations of associative neurons in the weighting matrix. In the case of the C4 function, there are twice as many clusters, since the signals of the input neurons are identical and it is possible to rearrange these neurons in the synapse matrix.

Each cluster in Figure 3 corresponds to a peak with a cut-off top in the fitness landscape. We emphasize that the quality of functioning (the level of fitness) of the structures shown in the figure is identical, which follows from the nature of functionally invariant transformations and is confirmed in a computational experiment.

From the comparison of the left and right 3D images, a useful methodological conclusion can be drawn that representations of the properties of the fitness landscape critically depend on which structural elements are taken into account. In this case, 8 and 16 structural clusters appear only with a certain selection of parameters (weight coefficients) of the NNMO. Additionally, the bi- and polymodality of frequency distributions of pairwise Cartesian distances between NNMO structures is one of the indicators of cluster structure existence (Bartsev, Bartseva, 2010).

As already noted, the initial weight coefficients of the NNMOs were set by a random number generator, and all NNMOs started from different starting points (near 0) in the structure space. Then the presented result suggests the possibility of different outcomes of evolution, but it does not say anything about the stability and reproducibility of the evolutionary trajectory

**Fig. 4.**
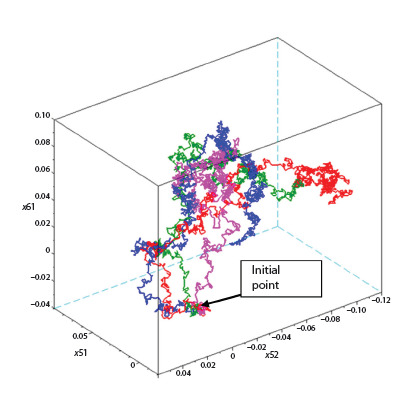
An example of the evolutionary trajectories of NNMOs for the A3 function in coordinates 25/26/31.

The reproducibility of the trajectory of simpler NNs for other tasks has been studied previously (Bartsev, Baturina, 2019). It has been shown that throughout the learning process, each point of the trajectory can generate a bundle of alternative trajectories, nevertheless directed towards the nearest cluster corresponding to maximum fitness. In this paper, to demonstrate the generally expected results, several implementations of training trajectories from the same initial state were obtained (Fig. 4). It is easy to see the rapid divergence of evolutionary trajectories, which is due: firstly, to the randomness of perturbations (mutations) of the weight coefficients; and secondly, to the non-reproducibility of the flow of events (Fig. 2) (the surrounding world) in which NNMOs were trained.

As noted above, only NNMOs consisting of 6 or more neurons can adapt (learn this task) to a given environment. To estimate the minimum number of synapses capable of performing a given function (in given case, A3 and C4), the NNMO complexity reduction procedure was used. This procedure consisted in the sequential removal of minimal (in modulus) synapses and subsequent retraining of the NN to a given quality of functioning.

**Fig. 5.**
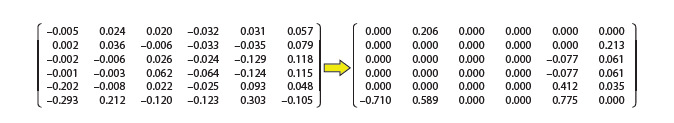
An example of reducing the complexity of a 6-neural NNMO with the A3 function

It was shown that the maximum number of weight coefficients for which the NNMO retains the same quality of functioning is 11 for sequence A3 (Fig. 5), and 13 for sequence C4. These differences in the minimum number of synapses are expected, since the C4 function is more complex than A3 due to the fact that the neural network needs to store the memory of the stimulus for longer before giving a response.

One more important point should be noted: if we take the structure of the reduced NNMO and replace the remaining non-zero synapses with random numbers (even conserving the sign), then NNMO learning does not occur. It follows from this that for learning (the evolutionary process), it is necessary to have degrees of freedom, i. e. weighting factors that are not fully involved in the implementation of the function and allow avoiding obstacles on the landscape of the fitness function via other dimensions.

**Fig. 6.**
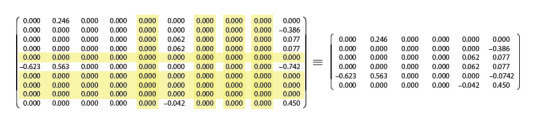
Transfer of the already reduced structure of 10-neuron NNMO (A3 function) to the 6-neuron configuration by deleting the zero rows (and the corresponding columns) of the synapse matrix.

It should be noted that the minimum number of synapses does not depend on the size of the initial neural network, but depends only on the function performed (Fig. 6). This fact allows us to assert that function complexity (for a given transient characteristic of a neuron) is an invariant quantifiable property of the function itself.

The NNMO reduction procedure can be interpreted as the price paid for a non-zero weighting factor. In this case, the formation of a reduced structure corresponds to the rigid specialization of the organism, which subsequently prevents it from breaking out of the evolutionary impasse.

The minimum number of synapses required for the functioning of NNMOs can be taken as an estimate of functional complexity. Then the total number of NNMO synapses can be considered as a potential complexity, which is similar to the information capacity of the class (Eigen, 1971).

It follows from computational experiments that in order for the evolutionary process to be carried out, a redun- dancy of potential complexity over functional complexity is necessary, and this redundancy leads, in turn, to a multiplicity of evolutionary outcomes, which is possible only when the same function can be implemented by different structures.

## Discussion

The results of the study of the heuristic evolutionary model have shown that, on the one hand, with a significant redundancy of potential complexity over functional complexity, it is impossible to predict the final structure of the NNMO. But, on the other hand, the introduction of restrictions, for example, the requirement of a minimum number of synapses, leads to a decrease in the number of possible final variants of the structure – their almost complete coincidence is possible, even with a difference in the initial size of the NNMOs. For example, comparing the right-hand weighting matrices in Figures 5 and 6, it is easy to see that the matrices of reduced 6- and 10-neuron NNMOs are very similar – it is enough to swap the bottom two rows and change the signs of synapses in the rows, that is, to make discrete functionally invariant transformations of the structure.

The conclusions by the heuristic model (from 2005) about the potential multiplicity of evolutionary outcomes were illustrated in the works (Podgornaia, Laub, 2015; Starr et al., 2017), where it was shown that in addition to the natural set of four amino acids, about 1 % of the combinations from the total set of 160 thousand can perform their function (of the same quality) in both proteins considered. At the same time, in the sequence space, the studied region splits into subdomains (clusters), within which evolutionary movements can occur relatively freely, while transitions between clusters are difficult, which corresponds well to the heuristic neural network model.

Herewith, if there are limitations and if the maximum of the fitness function is found in the vicinity, evolutionary trajectories can be reproduced (Weinreigh et al., 2006; Meyer et al., 2012; Orgogozo, 2015), which also agrees with the heuristic model using the example of reduced NNMOs.

Note that the results obtained are not purely heuristic and computationally experimental. The possibility of functionally invariant transformations leads to the fact that “mutations” of the structure with identical or very similar quality of functioning (equal or close fitness) are possible, which corresponds to neutral mutations of Kimura (1979). The consequence of this is that at the next step, the evolutionary trajectory may take several paths equivalent in terms of fitness function.

In summary, we can agree with the claim that mathematical models (Garte et al., 2025) are generally unable to predict a specific evolutionary trajectory. Apparently, the requirement to predict a specific trajectory is in most cases an incorrect request

Instead, the model, working with ensembles of trajectories, can calculate the probabilities and conditions of an evolutionary outcome, which will provide an understanding of the general properties of evolving systems. For example, the computational construction of the Agekyan–Anosova “homological map” (Lento et al., 2008) showed that the dependence of the lifetime of a 3-body system on the initial state has a fractal structure – and it becomes clear what can be expected from this system in principle.

## Conclusion

An abstract heuristic model of evolution based on general assumptions about the nature of living things allows us to formulate assumptions (hypotheses) about the general properties of evolving systems. In particular, the very possibility of evolutionary change depends on the redundancy of the structure over the complexity of the function under selection, which generally leads to a potential multiplicity of evolutionary outcomes due to the ambiguity of structuralfunctional relationships. That is, in the general case, the specific path and the final structure (and its very achievement) of an evolving system are not deterministic given the functional equivalence of the evolutionary outcomes.

## Conflict of interest

The authors declare no conflict of interest.
